# Development of Wide-Angle Short-Wave Pass Thin Film Based on the Ultra-Thin Silicate Glass

**DOI:** 10.3390/ma15134706

**Published:** 2022-07-05

**Authors:** Xiuhua Fu, Suotao Dong, Shifu Xiong, Cheng Li, Xiaodong Chen

**Affiliations:** 1 School of Optoelectronic Engineering, Changchun University of Science and Technology, Changchun 130013, China; fuxiuhua@cust.edu.cn; 2 Institute of Functional Crystals, Tianjin University of Technology, Tianjin 300382, China; xsf_optics@email.tjut.edu.cn; 3 School of Energy and Power Engineering, Changchun Institute of Technology, Changchun 130103, China; li.cheng@ccit.edu.cn; 4 Changzhou Xinli Ion Technology Co., Ltd., Changzhou 213017, China; engineer@xinli-ion.com

**Keywords:** laser medical treatment, wide-angle, cathode sputtering, short-wave pass filter film, film peeling, hundreds grid fastness test

## Abstract

With the rapid development of laser medicine, there are higher requirements placed on the performance of optical components in various medical systems. This paper is aimed at exploring the critical optical devices of medical equipment for treating periodontitis and gingivitis. The cathode sputtering method was used to produce the wide-angle short-wave pass filter, and a hundreds grid fastness test was conducted to detect the occurrence of film peeling. Considering the results of SEM, transmission spectrum, and stress test of the sample, an analysis was conducted as to the cause of poor bonding force for the film. By increasing the amount of argon gas and adjusting the baking temperature, the problem of film peeling was resolved. Besides, a short-wave pass filter film with good bonding and low roughness was obtained to meet the requirements of laser medical equipment.

## 1. Introduction

### 1.1. Laser Chemical

With the progress of modern science and technology, the innovation of laser technology has penetrated various fields, such as science and technology, medical treatment, military affairs, and our lives. Among them, laser medical technology is developing rapidly, and it has achieved different degrees of success in both external laser surgery and internal laser ablation [1]. Since the world’s first ruby laser was launched in 1960, this new light source and the resulting new laser technology have been applied to the medical field. Laser is known as the “light of life”. As a new treatment method in clinical treatment, laser therapy has the advantages of being simple, fast, and causing more minor trauma, which could not be said of traditional treatment methods. Laser medical technology has been applied for more than forty years. The combination of laser and oral treatment began in 1964 [2]. With the development of laser technology, it is more and more widely used in the field of oral medicine. The practicability and safety of lasers in the oral cavity area have been certified in many aspects and have been approved by the U.S. Food and Drug Administration. The types of lasers used in oral clinics mainly include the semiconductor laser, CO_2_ laser, argon ion laser, Nd: YAG laser, Er: YAG laser, and Er: YSG laser. The principle of the laser treatment of oral diseases is mainly to produce a conditioning effect, reduce inflammation and pain, fight infection, improve blood circulation, and enhance immune function [3]. It can also improve the microcirculation system, inhibit smooth muscle tension, increase capillary blood flow, enhance antithrombin activity coefficient, increase tissue permeability, reduce hematoma, inhibit traumatic tissue, increase RNA and DNA production, produce single-molecule oxygen, play an essential role in cell regeneration, restore pathological degradation caused by nerve injury, and enhance the bactericidal effect of blood cells. It can eliminate inflammation and pain, eliminate infection, promote the absorption of exudate and accelerate wound healing. When a laser beam irradiates biological tissue, it can produce photoelectric, magnetic, photothermal, physical, and photochemical radiation. The laser can use these characteristics and effects for coagulation, cutting, burning, gasification, acupuncture, analgesia, anesthesia, and other functions. Lasers can be divided into high-energy lasers and low-energy lasers according to different energy levels [4]. A high-energy laser refers to a laser with a power of more than a few watts, which may have an irreversible effect on the body. It is often used to vaporize and cut the focus. A low-energy laser usually refers to a laser with milliwatt power, which primarily functions by exerting its biological stimulation effect. The low-energy laser can directly irradiate biological tissue without irreversible damage, so it is more widely used in non-invasive medicine. While laser medical equipment is widely used, its safety should also attract the attention of manufacturers and users. In particular, light radiation has been declared the fourth largest environmental pollution source by the World Health Organization and has become an invisible “killer” endangering human health. The harm of laser radiation is mainly reflected in the damage to the eyes and skin [5].

Photobiological hazards can be roughly divided into photochemical hazards and thermal hazards according to different damage mechanisms. The photochemical effect refers to the photochemical reaction caused by light irradiation. This reaction is a unique chemical reaction of the excited molecular state. Medium dose ultraviolet radiation and short-wavelength light irradiation for a long time may cause irreversible changes in some biological tissues, such as skin, eye crystals, and especially retinal membrane, resulting in organ damage [6]. The thermal effect refers to when biological tissue absorbs enough irradiation energy, the cells in the irradiation area will burn. The cornea has a strong absorption ability of ultraviolet radiation less than 315 nm for human eyes. This ultraviolet radiation may cause keratitis, conjunctivitis, cataract, etc. The lens behind the cornea can absorb ultraviolet radiation with a wavelength of 315 ~ 400 nm, although its absorption by the cornea is weakened, which can also cause damage such as keratitis and conjunctivitis. Near-infrared light with a wavelength range of 850 ~ 1100 nm can pass through the cornea and lens and reach the retina, which may cause a retinal burn. This band is also known as the retinal hazard area. Simultaneously, the near-infrared band greater than 850 nm can go deep into the dermis and sub-dermis of the skin, resulting in skin burns [7]. At the same time, there has been a well-developed suite of laser applications in dental medicine. However, dental care belongs to the category of medical aesthetics. Patients have higher requirements for aesthetics, and the technical requirements for doctors are also very high. Therefore, providing doctors with an excellent surgical vision has become the most challenging problem in medical equipment manufacturing. In 1994, Zhou Yuecheng and others of Chengdu Laser Medical Research Centre [8] gave a brief description of the application of oral laser treatment equipment. However, to this day, there is no adequate protection against the laser’s harm to the surgeon.

With the advancement of science and technology, physical technologies such as optoelectronics continue to mature in the oral field. Lasers have created a new area for diagnosing and treating oral diseases through various biological stimulation effects such as light, electricity, and heat, making many tedious diagnoses and treatments of oral diseases [9]. It is simple and highly effective; it fulfills the need for minimally invasive, preservation, and comfortable oral treatment, especially for patients with “dental phobia”. The laser may become a new type of painless and noiseless treatment tool to assist or even partially replace traditional pneumatic drills and surgical instruments, providing a new diagnosis and treatment direction for the treatment of diseases [10]. A laser can be used in many branches and fields, including teeth, periodontal, oral mucosal disease, orthodontics, oral prosthodontics, oral and maxillofacial surgery, and oral and maxillofacial beauty. It requires clinicians to use laser-related physics knowledge flexibly based on mastering solid oral professional knowledge and using the laser correctly, otherwise, there will be abuse or even counterproductive effects. Lasers are still being updated and developed continuously, and doctors in various dental professions are trying to implement the use of lasers [11,12].

Laser dentistry has been around since the 1990s. It is a minimally invasive, effective, safe, and less painful alternative to traditional dental procedures. Laser dentistry can treat various dental conditions, such as cavity detection and removal [13]. Lasers cannot remove pre-existing restorations, such as dental crowns and fillings. Lasers can treat many dental conditions affecting the soft tissues (gums) and hard tissues (teeth and bones). They can also remove benign tumors, regenerate nerves, reduce inflammation, relieve jaw pain, and even whiten teeth. The benefits of laser dentistry include less pain and discomfort, and a faster healing time, with only minor damage to the gums and teeth [14,15]. There is also a reduced need for sutures and local anesthesia. The downsides of laser dentistry are that it is generally more expensive than traditional dental procedures, and not all dental procedures can be completed with lasers. Similar to conventional methods, insurance partially covers most therapeutic laser treatments. Insurance never covers cosmetic procedures [16]. Therefore, the better completion of laser protection work is an essential basis for the development of laser dentistry. The protective goggles prepared in this paper are devices of great significance to both healthcare workers and patients. The device is realized by preparing Nb_2_O_5_ and SiO_2_ thin films by cathode sputtering. The reasons for the selection will be explained in detail later.

### 1.2. Cathode Sputtering

When charged or neutral particles with sufficient energy collide with the surface of an object, they can transfer energy to the atoms on the surface. As long as the energy obtained by the surface atoms is more significant than their ionization energy, they can get rid of the surrounding atoms’ shackles and leave the object’s surface. This phenomenon is called sputtering. Glow discharge occurs when a high voltage is applied between two electrodes in a vacuum of several hundred Pascals [17]. The glow discharge phenomenon occurs when DC voltage is applied. The characteristics of the glow discharge phenomenon are: the distribution of the glow is not uniform and can be divided into eight regions; most of the voltage drop falls in the Crooks dark region; each region changes with the change of vacuum and current, and also changes with the change of vacuum degree and current-differences in the distance between the poles. Cathode sputtering is a phenomenon of DC glow discharge. When the vacuum degree in the vacuum chamber is 13 Pa, a specific voltage is applied between the anode and cathode electrodes, and a self-excited release occurs in the gas. The atoms or groups of atoms emitted from the cathode can be deposited on the anode or the vacuum chamber wall. The cathode sputtering discharge circuit is formed by the movement of positive ions generated by gas discharge towards the cathode and the movement of primary electrons towards the anode. Supplement the consumption of one electron to maintain. Therefore, the discharge measured in the external circuit is the sum of the current of positive ions bombarding the target and the current of secondary electrons emitted by the cathode [18].

### 1.3. Silica Thin Film

This paper studies a protective goggle used in dental laser medical equipment and solves the adverse consequences of damage to the human body caused by a part of the light. Due to equipment entry into the population, Nb_2_O_5,_ and SiO_2_, two commonly used visible light and near-infrared materials, were selected for comprehensive material safety considerations. However, some problems were encountered during the evaporation process, which will be explained later. First of all, we want to understand the optical properties and stress of SiO_2_ film to prepare SiO_2_ film for subsequent evaporation correctly [19].

Silica is an optical thin-film material with positive insulation, corrosion resistance, and a high laser damage threshold. Therefore, it is widely used in semiconductor technology. With the continuous development of semiconductor technology and the universal application of high-power semiconductor lasers, the requirements for the preparation process of thin films are becoming more and more strict [20]. SiO_2_ thin films are mainly prepared by plasma-enhanced chemical vapor deposition, magnetron sputtering, and ion beam evaporation. SiO_2_ thin films deposited by PECVD have the advantages of low reaction temperature, uniform film formation, complete step coverage, and process repeatability. However, large lattice mismatches or residual impurity defects between the film and the substrate structure lead to film stress generation [21,22]. The existence of stress also has a certain impact on the device’s performance, which makes researchers begin to pay attention to the measurement and research of thin-film stress. The stress mechanism of SiO_2_ film has been studied thoroughly, and the content is mainly divided into three categories: thermal stress, and internal and external stresses. The thermal expansion coefficient of materials is different in different situations [23]. The film-forming process is generally carried out at high temperatures (>200 °C). When the high temperature is restored to normal temperature, the deformation between the substrate and the film is different, resulting in the deformation of the interface lattice and the film stress. The second point is that film stress is caused by the formation of a unique microstructure in film growth. The last one is the stress change caused by environmental factors. When the film is stored in different environments, the film stress changes and the effects of temperature, humidity, and air pressure atmosphere are more obvious [24].

The film stress is closely related to the film structure. Generally, the film stress comes from the incomplete structure (impurities, vacancies, dislocations, etc.) formed in the film growth process, the existence of surface states, and the lattice difference between the film and the substrate, resulting in mismatch and lattice deformation. After the film is formed, the film stress can be changed by changing the film structure [25,26,27]. Heat treatment is the most common way to change the film structure because the high temperature of heat treatment can rearrange the atoms in the film and reduce or eliminate the defects in the film [28]. It can also change the lattice deformation at the interface between the film and the substrate to change the film stress [28]. Therefore, in the subsequent experiments, to solve the problem of SiO_2_ spalling, this paper also adopts heat treatment to rearrange the atoms [29].

The stress of the film is mainly composed of surface tension [30], thermal stress, and internal stress. The surface tension of the solid surface is about 10^−2^ N/cm^2^, 10^−3^ N/cm^2^, while the stress of the dielectric film is generally in the order of 104 N/cm^2^, so although the surface tension is a kind of stress, in most cases, the film stress is of the order of magnitude. Contributions may not be counted. The thermal stress and internal stress are very complex. They are related to the test conditions of the film stress, the use conditions of the film element, the temperature during coating, the evaporation rate of the coating material, the amount of oxygen in the SiO_2_ coating, the thickness of the film, and the thickness of the film before coating. The basic vacuum degree and the annealing treatment after coating have a lot to do. The test conditions that affect the film stress are temperature and humidity [30]. If the temperature and humidity are unstable, the film stress varies greatly. Therefore, the temperature of the room where the interferometer is located before and after the coating is controlled at around 23 °C, and the humidity is the same before and after coating. The stress of thin-film elements is very different when used in a vacuum and in the atmosphere. Generally speaking, the tensile stress of the thin-film element increases after being placed in a vacuum. The substrate surface needs an inspection before coating, and the film should be measured in a vacuum condition as well if deposition processed in a vacuum. The author assumes then, that the final use condition is the atmosphere, so the reflected wavefront of the high-reflection film after coating and the reflected wavefront of the substrate before the coating is conducted in the atmosphere [31]. The temperature has a huge influence on the thermal stress of the film. The thermal stress is mainly caused by the difference in thermal expansion coefficient between the film layer and the substrate. So, if the temperature during coating is the same as the basic temperature during measurement, the thermal stress of the film is very small, which is called the Cold plating scheme. The size of thermal stress can be adjusted by selecting the appropriate coating temperature. However, the temperature also directly affects the internal stress because the internal stress mainly depends on factors such as the microstructural defects of the film. The temperature has a direct effect on the microstructure of the film [32]. Now, there is a clearer point of view: for dielectric films such as HfO_2_, the internal stress decreases with increasing temperature. From the influence of temperature, a parameter, the calculation of the total stress of the film is very complicated. The coating material’s evaporation rate generally affects the film’s internal stress. The current theory holds that the changes in the microscopic defects of the film, such as recrystallization and film phase transition are related to the evaporation rate. However, the relationship between the magnitude of the internal stress of the film and the evaporation rate is now derived from published data. There is no rule which may be related to the material of the specific dielectric film. It is believed that increasing the amount of oxygen in SiO_2_ plating can reduce the internal stress of SiO_2_, and it is generally believed that the internal stress of SiO_2_ is expressed as compressive stress [33]. The thickness of the film layer also directly affects the internal stress of the film because the thickness of the film layer is closely related to the recrystallization of the film. This close relationship is related to the specific dielectric film material and other factors. It is not simply that the thicker the film layer, the greater the internal stress of the film. The basic vacuum degree before coating also influences the internal stress of the film because the residual in the vacuum enters the film, and the crystal structure of the film deviates from its bulk material. More seriously, the difference in the basic vacuum degree before coating may change the internal stress of the film from tensile stress to compressive stress. The annealing treatment after coating can change the microscopic defects of the film, so it has a great relationship with the internal stress of the film, but changing the microscopic defects of the film does not necessarily mean reducing the internal stress of the film. Still, it may also increase the film’s internal stress [34].

The protective glass is researched, and a laser protective filter film that cuts visible light through the near-infrared band is designed and prepared [35]. The space in the oral cavity is limited, and the equipment has strict restrictions on the thickness of the filter. Moreover, oral surgery requires the doctor to have an adaptable field of vision from a vertical angle, and the doctor’s assistant who observes at a large angle also requires a good field of vision. However, the film stress generated by the thinner substrate is more obvious, causing stress problems, so the film also peels off [36]. In this paper, the spalling problem on the thin substrate is studied, and the short-wave pass filter protective film with wide-angle and high transmittance is prepared.

## 2. Experiment

### 2.1. Material

According to the requirements of the laser medical system, the technical parameters of the short-wave pass filter are shown in Table 1.

Due to the limitation of the size and thickness of the filter, the single-sided transmittance of 410 nm-810 nm has a requirement of as high as 94%. D263 T borosilicate glass, which is thinner and has high visible light transmittance, is used as the filter. The substrate, with a thickness of 0.21 mm, is a low-roughness fire-polished surface, impervious to moisture, and high light transmittance glass that is easy to cut.

**Figure 1:**
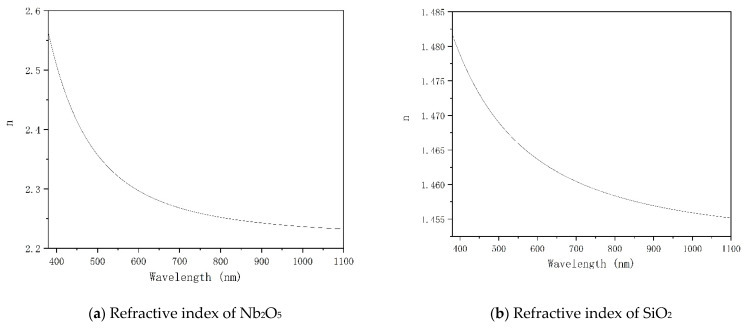
Refractive index of two materials.

When the material’s photon energy is more incredible than the forbidden bandwidth, the electronic transitions from the valence band to the conduction band to produce intrinsic absorption. Generally, the intrinsic absorption of transparent materials is small. Under the premise of meeting the requirements of the spectrum and the requirements of non-toxic and harmless dental medical devices, the commonly used high refractive index materials are Nb_2_O_5_, TiO_2_ and Ta_2_O_5_. TiO_2_ is difficult to maintain in a stable state for a long time, usually in a mixed form of TiO_2_, Ti_2_O_3_, TiO, and Ti, so the refractive index is relatively unstable. Ta_2_O_5_ is easy to produce the porous film, which does not meet the requirements of the ring test. Therefore, Nb_2_O_5_, which has a relatively stable refractive index and is suitable for preparing short-wavelength filter films, is used as a high refractive index material. SiO_2_, MgF_2,_ and Al_2_O_3_ commonly use low refractive index materials in this band. MgF_2_ has a low refractive index, large tensile stress, and a poor combination of Nb_2_O_5_. The refractive index of Al_2_O_3_ is higher, the number of layers needs to be more, and the emission of impurities is more significant. Therefore, SiO_2_, which has a moderate refractive index and is suitable for preparing short-wavelength filter films, is selected as the low refractive index material. The refractive indices of Nb_2_O_5_ and SiO_2_ are shown in Figure 1.

### 2.2. Film Stack Design

(0.5LH0.5L) S has better short-wave pass characteristics, so we use this film system [pqp]. Its characteristic matrix is [4]

(1) $$M_{\mathrm{pqp}}=\left[\begin{array}{cc} \cos\delta_{p} & \left(i\sin\delta_{p}\right)/\eta \\ i\eta_{p}\sin\delta_{p} & \cos\delta_{p} \end{array}\right]\cdot \left[\begin{array}{cc} \cos\delta_{q} & \left(i\sin\delta_{q}\right)/\eta_{q}\\ i\eta_{q}\sin\delta_{q} & \cos\delta_{q} \end{array}\right]\cdot \left[\begin{array}{cc} \cos\delta_{p} & \left(i\sin\delta_{p}\right)/\eta_{p}\\ i\eta_{p}\sin\delta_{p} & \cos\delta_{p} \end{array}\right]={\left[\begin{array}{cc} M_{11} & M_{12}\\ M_{21} & M_{22} \end{array}\right]}^{\;}$$

In the formula, $\delta_{p}$ and $\delta_{q}$ are the effective phase thicknesses of the *p* and *q* layers, respectively, and $\eta_{p}$ and $\eta_{q}$ are the equivalent refractive indexes of the *p* and *q* layers, respectively [32].

The characteristic matrix of the symmetric membrane system has the same properties as the single-layer membrane so that it can be replaced by a single-layer particular equivalent membrane system, namely:

(2) $$M=\left[\begin{array}{cc} M_{11} & M_{12}\\ M_{21} & M_{22} \end{array}\right]\;\;=\left[\begin{array}{cc} \cos\Gamma & \left(i\sin\Gamma \right)/E^{-}\\ iE\sin\Gamma & \cos\Gamma \end{array}\right]$$

Γ is

(3) $$\Gamma =\cos^{-1}M_{11}=\cos^{-1}\left[\cos2\delta_{p}\cos\delta_{q}-0.5\left(\frac{\eta_{p}}{\eta_{q}}+\frac{\eta_{q}}{\eta_{p}}\right)\sin2\delta_{p}\sin2\delta_{q}\right]$$

It can be seen that the characteristic matrix *M^S^* of the periodic film system (PQP)^S^ should be the product of the basic periodic characteristic matrices:

(4) $$M^{S}={\left[\begin{array}{cc} \cos\Gamma & i/E\sin\Gamma \\ iE\sin\Gamma & \cos\Gamma \end{array}\right]}^{S}$$

*S* is cycle

(5) $$M^{S}=\left[\begin{array}{cc} \cos S\Gamma & i/E\sin S\Gamma \\ iE\sin S\Gamma & \cos S\Gamma \end{array}\right]$$

Equation (5) shows that the symmetric periodic film has an equivalent refractive index E_S_ in its transmission band and the equivalent phase thickness $\Gamma_{S}$ is equal to *S* times the equivalent phase thickness of the fundamental period [37].

The boundary of the cut-off zone is determined by $M_{11}=-1$:

(6) $$\Delta \lambda =2\Delta g\lambda_{0}=\frac{4\lambda_{0}}{\pi }\sin^{-1}\left|\frac{n_{H}-n_{L}}{n_{H}+n_{L}}\right|$$

Among them, Δλ is the bandwidth, $\lambda_{0}$ is the center wavelength, and $n_{H}$ and $n_{L}$ are the refractive indices of the high and low refractive index materials respectively.

This paper chooses Sub|(0.5LH0.5L) ^s^| Air as the basic film system. Sub represents the base material silicate glass, H represents the high refractive index material Nb_2_O_5_, L represents the low refractive index material SiO_2_, s represents the number of cycles, and Air Represents air. According to Formula (6), the larger $n_{H}-n_{L}$ is, the wider Δλ is. According to the parameter requirements, at the center wavelength of the cut-off band at 975 nm $n_{H}$ = 2.24197, $n_{L}$ = 1.4561, substituting into Equation (6), the cut-off bandwidth is Δλ ≈ 268.96 nm, and the cut-off bandwidth is 250 nm according to the design requirements, so the selection of claim. Use Essential Macleod to optimize the film layer. After setting the film system goal, use Needle synthesis (pin insertion method) to optimize the film system. Then adjust the thickness of the film system. The film structure and film thickness are shown in Table 2.

**Figure 2:**
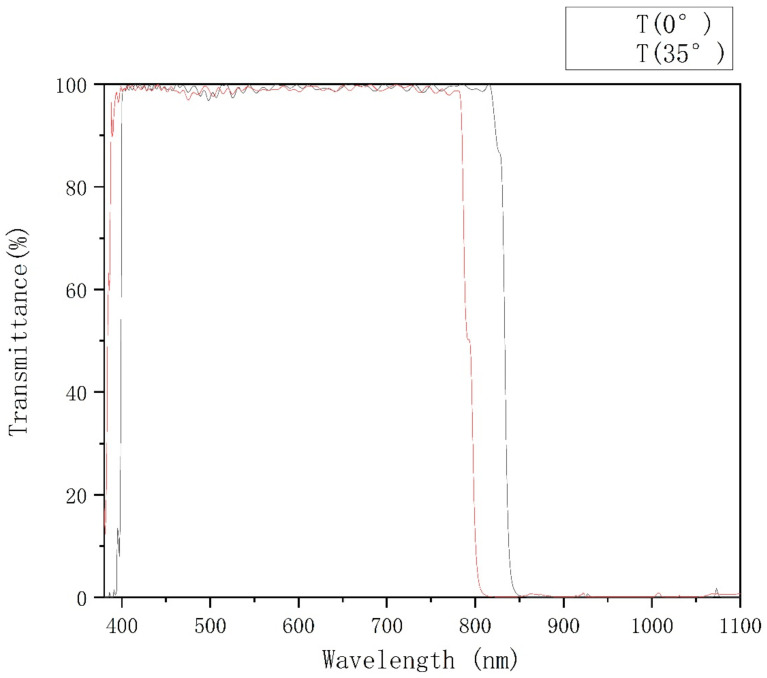
Design Spectral to 0° and 35°.

The theoretical design transmittance spectrum is shown in Figure 2. When the incident angle is 0°, the average transmittance of 410 nm ~ 810 nm is 98.97%, and the minimum transmittance T_min_ = 97.63%, 850 nm ~ 1100 nm, without considering the back reflection. The average transmittance is 0.16%; when the incident angle is 35°, the average transmittance of 410 nm ~ 775 nm is 98.88%, and the minimum transmittance T_min_ = 97.03%, the average transmittance of 830 nm ~ 1100 nm is 0.18%, meeting the technical parameter requirements. The back surface is prepared with an anti-reflection coating with an incident angle of 0°, 410 nm ~ 810 nm, and 35°, 410 nm ~ 775 nm. The average transmittance is 99.4%. According to the specific requirements of the core components of the laser therapeutic instrument, the spectral requirements shall be met from 0 to 35 degrees. The handheld device is usually held by a doctor’s assistant, which must not interfere with the doctor’s other medical behaviors, but also conform to his own line of sight, so it needs 0 to 35 degrees. According to the principle of polarization spectroscopy, the direction of spectral movement from 0 to 35 degrees is the same. Therefore, only two points of 0 to 35 degrees are required to meet the requirements of all angles within the range of 0 to 35 degrees.

### 2.3. Preparation Methods

This experiment was performed on the radiance cathodic magnetron sputtering equipment produced by Evatec. The equipment has five target positions and a capacitively coupled plasma ion source as additional deposition. Nb_2_O_5_ and SiO_2_ films were deposited by cathode sputtering by oxygenation with Nb target and Si target, respectively.

Put the ultrasonically cleaned substrate into the first-class vacuum chamber, and the robot arm transfers it to the second-class vacuum chamber. When the vacuum reaches 7 × 10^−5^, turn on the baking, heat up to 170 °C, and maintain it for 600 s. When the vacuum reaches 7 × 10^−6^, deposition starts on the silicate glass substrate, the power is 7500 W, and the argon content is 80 sccm. The thickness of the Nb_2_O_5_ monolayer film is about 500 nm; on the ZF6 substrate, the power is 5000 W and the argon content is 35 sccm. A single layer of SiO_2_ film with a thickness of about 1000 nm was prepared under these conditions. After measuring the spectrum, use the envelope method to calculate the optical constants as follows:

The short-wave filter film is prepared for the following test according to the designed film system.

## 3. Testing and Analysis

### 3.1. Firmness Tests and Analysis

**Figure 3:**
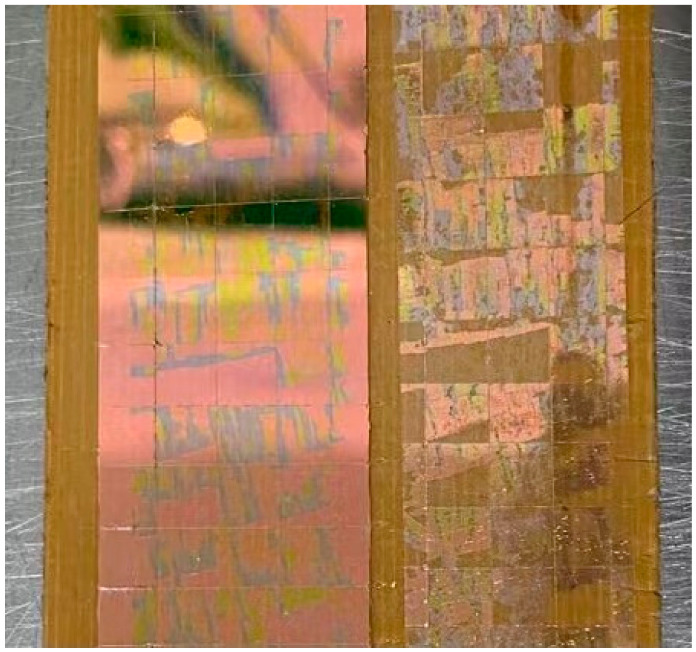
Peeling test.

The Adhesion Cross-Cut Test [38] methods are used to test the film’s firmness. The specific content is as follows: The wafer is cut into a size of 25 mm × 100 mm, and then 5 × 20 squares of the same size are drawn with a cutting knife. After fixing it, use 3M tape to pull the film. Observe the number of squares peeled off the film, and the film release rate. As shown in Figure 3, after the Adhesion Cross-Cut Test result, the film is released by more than 80%.

The film is formed on the surface of the substrate, so there will be a specific interaction between the substrate and the film, and this interaction is adhesion. When the film is attached to only one surface of the substrate, the substrate and the film are mutually constrained, resulting in easy strain in the film. If any cross-section perpendicular to the membrane surface of the film is considered, an interaction force will be generated on both sides of the cross-section. This interaction force is called internal stress. Internal stress, also known as intrinsic stress, mainly depends on factors such as the microstructure and defects of the film [39]. The main one is the interaction between the grain boundary and the film spacing and the mismatch of the substrate lattice. Hoffman [40] and others proposed a model where the internal stress is related to the elastic stress between crystal grains generated during the growth and merging of crystal nuclei. Corresponding to the internal stress is the external stress. After the coating is completed, the film’s physical environment (working pressure, humidity, etc.) will change. When it is different from the original conditions, it will cause stress, called external stress. Coexisting with intrinsic stress and external stress, temperature changes also cause thermal stress. For the cathode sputtering method to prepare Nb_2_O_5_ and SiO_2_ films, the factors affecting stress include temperature, pressure, Setpoint value (argon-oxygen ratio), argon charge, and ion source parameters. This article discusses two important aspects of temperature and argon charge [41].

### 3.2. Firmness Tests and Stress Tests

**Figure 4:**
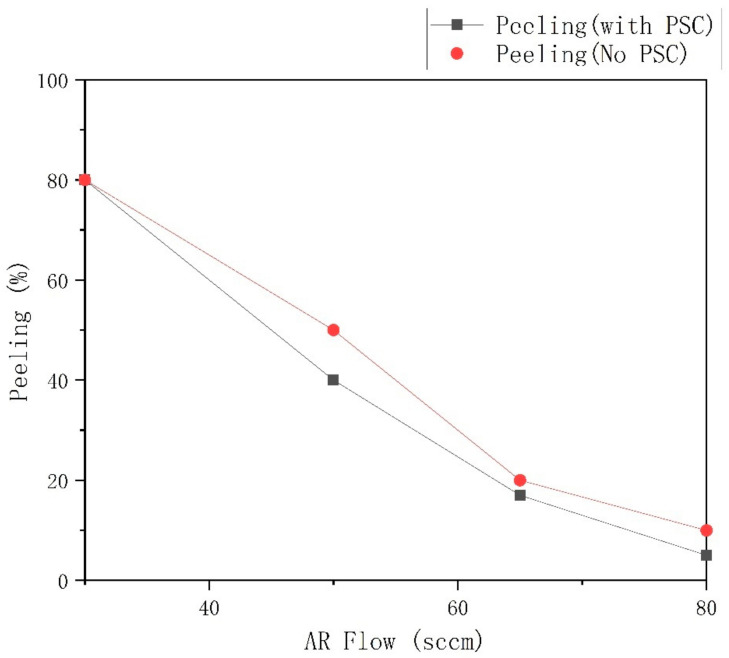
Peeling test results with Different Argon flow for SiO_2_.

Regarding whether there is an ion source, the aeration amount and the amount of Ar Flow of the Si target are adjusted. The test results are shown in Figure 4.

As shown in Figure 4, between 35 sccm and 80 sccm, the overall film release rate without an ion source is significantly lower than that of the ion source. Without the aid of an ion source, with the continuous increase of argon from 35 sccm to 80 sccm, the film release rate gradually decreases from 80% to 10%. If the argon gas charge exceeds 80 sccm, the vacuum degree will be lower than 7 × 10^−5^. The mechanism of cathodic magnetron sputtering is essentially to ionize the charged argon gas into a plasma state. Under the combined action of an electric field and a magnetic field, the argon ions bombard the target material, causing it to sputter onto the substrate surface to form a thin film. Therefore, the amount of argon directly determines the argon ion density, thereby affecting the sputtering degree of the target. Although the increase in argon ions can make the target react more fully, the remaining free argon molecules and argon ions will remain in the film, causing defects in the film. Therefore, the amount of argon requires an equilibrium value. In many experiments, a value with a sufficient reaction and only a few residual argon molecules are found, which is 80 sccm. Under this argon content, the reaction rate of the niobium oxide monolayer film is the fastest, which is 0.1218 nm/s, and the argon content measured by the residual gas analyzer is relatively small. The film glass phenomenon is also obvious, as shown in Figure 4.

**Figure 5:**
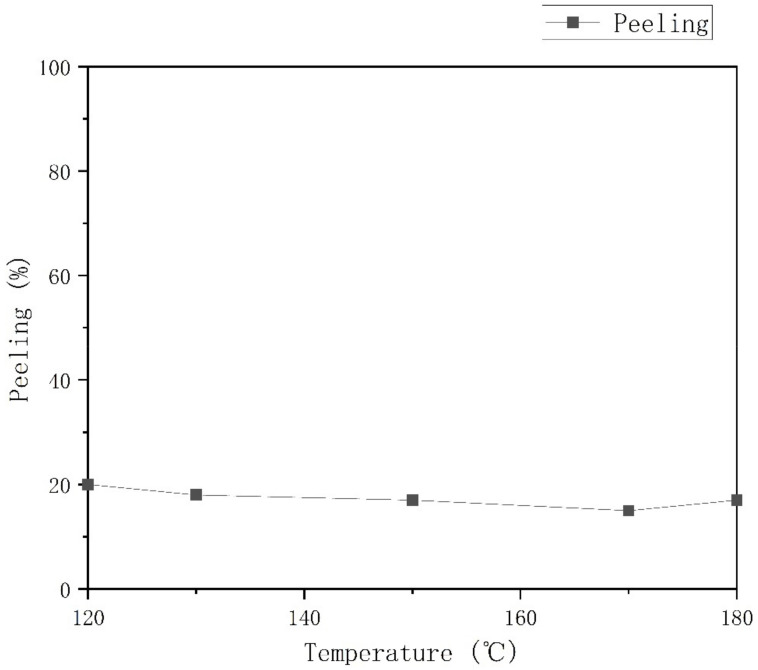
Peeling test results with Different Temperatures.

In the thermal stress part, under the premise that other parameters do not change, adjust the baking temperature and use the two methods of Temperature Fix (temperature adjustment) and Power Fix (power adjustment). The power control method is better than the former, and the effect of different temperatures on the film peeling phenomenon is shown in Figure 5.

As shown in Figure 5, as the temperature increases from 120 °C to 180 °C, the film release rate gradually decreases from 20% to 10%, then increases from 10% to 18%. It can be seen that within a certain range, the higher the temperature, the greater the thermal stress. Because the thermal expansion coefficients of the two materials are different, the stress is closer so that the film peeling phenomenon can be improved.

Through the comparison of the peel test, it is found that the argon gas charge of the silicon target has a greater influence on the film release phenomenon. The film stress of this part of the experiment was tested and analyzed. The test of the film stress is based on the Stoney formula, and the laser beam method is used to measure the substrate strain to calculate the stress. The sputtering of the film on the substrate causes the deformation of the substrate, and the stress causes the deformation of the substrate in the film. The curvature of the substrate after deformation will change, and the film stress can be obtained by measuring the changes in curvature before and after the substrate.

The expression of Stoney’s formula [42] is

(7) $$\sigma =\frac{E_{S}h_{S}^{2}}{6rh_{f}\left(1-v_{S}\right)}$$

In the formula, σ is the stress for substrate, r is Radius of curvature, $h_{f}$ is the thickness of the film, $E_{S}$ and $v_{S}$ are the Young’s modulus and Poisson’s ratio of the substrate, respectively.

**Figure 6:**
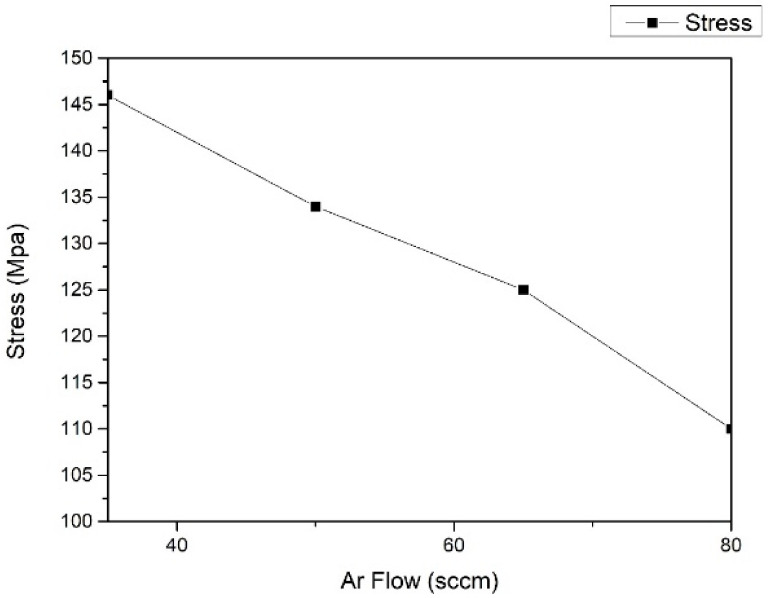
Nb_2_O_5_/SiO_2_ multilayer thin-film stress results.

As shown in Figure 6, the stress of argon gas is gradually decreasing from 35 sccm to 80 sccm. When the argon gas charge is 80 sccm, the stress is the smallest, 110 Mpa.

### 3.3. Scanning Electron Microscope Tests and Analysis

**Figure 7:**
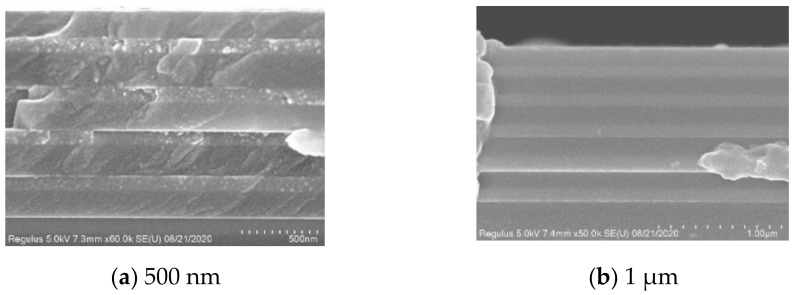
Nb_2_O_5_/SiO_2_ multilayer thin film SEM data for 35 sccm Ar.

Another part of the samples was selected for scanning electron microscopy to study further. The test results are as followings in Figure 7.

By observing the test results of the scanning electron microscope and comparing the test results of the two films, it is found that there are obvious defects in the SiO_2_ film in Figure 7 when the test result is filled with less argon gas (35 sccm). In cathode sputtering, Ar is introduced to maintain a certain degree of vacuum. The power is turned on to cause abnormal glow discharge between the cathode and anode to form a plasma zone so that the electric field accelerates the positively charged Ar+ to bombard the target so that the target material is sputtering. The silicon ions generated by the argon ion bombardment of the target material combine with cations to form a silicon oxide film attached to the substrate. If the number of argon ions is small, the mean free path of ion movement is smaller, and the number of silicon ions generated will also decrease. Therefore, the sufficiency of the contact between the argon ions and the target is particularly important. Therefore, the defects in the SiO_2_ film are most likely caused by insufficient bombardment and collision of the argon ions with the target.

**Figure 8:**
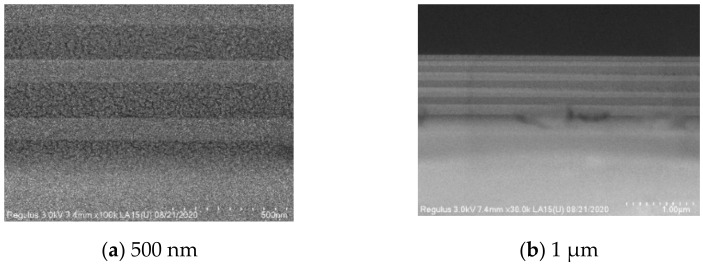
Nb_2_O_5_/SiO_2_ multilayer thin-film SEM data for 80 sccm Ar.

In the experiment, choose to increase the argon gas filling volume so that the argon ions fully contact the reactive particles to form a silicon oxide film with intensive density and less stress. Figure 8 is the SEM image measured after increasing the argon charge to 80 sccm, and the defect is eliminated.

### 3.4. Spectral Test

**Figure 9:**
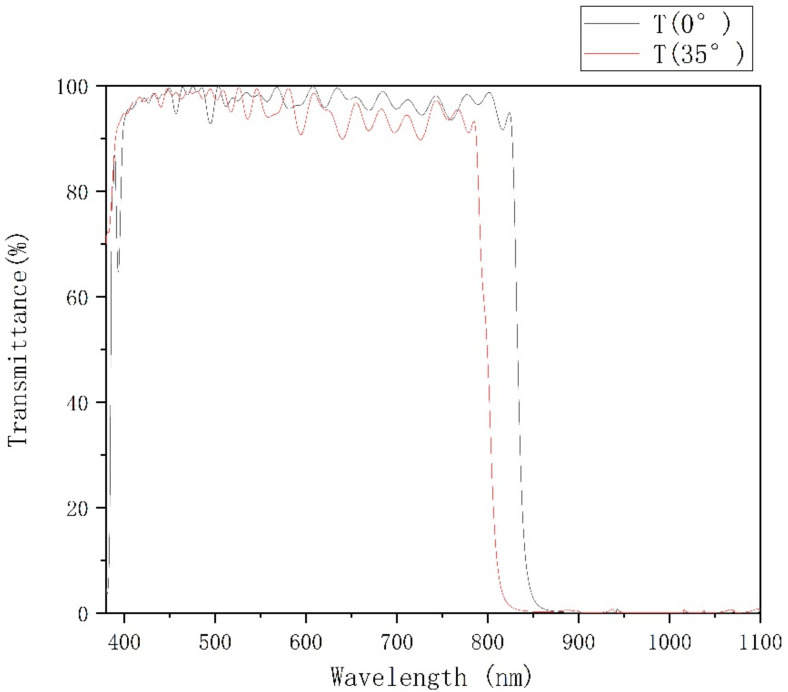
BPF Spectral test results for 0° and 35°.

After optimizing the argon filling and baking temperature parameters, the experiment was carried out again. Cary 6000 spectrophotometer produced by Agilent was used to test the test samples. After the front surface is coated with a single-sided coating, the back surface is coated with an anti-reflection coating. The transmittance test curve at 0° and 35° incident angles are shown in Figure 9.

When the incident angle of the double-sided coating is 0°, the average transmittance of 410 nm ~ 810 nm is 94.52%, the average transmittance of 410 nm ~ 810 nm is 98.82%, the minimum transmittance T_min_ = 97.41%, and the average transmittance of 850 nm ~ 1100 nm is 0.13%; When the incident angle is 35°, the average transmittance of 410 nm ~ 775 nm is 98.79%, the minimum transmittance T_min_ = 97.14%, and the average transmittance of 830 nm ~ 1100 nm is 0.17%, which meets the requirements of the filter film parameters.

## 4. Results and Discussion

Thin-film stress is mainly divided into two categories: growth stress and extrinsic stress. The growth stress due to bulk growth is mainly caused by surface interface stress, cluster merger, grain enlargement, vacancy annihilation, grain boundary void shrinkage, impurity merger, etc. According to the SEM image analysis, it can be seen that this paper’s experiment suffered due to insufficient gas to cause point defects in the SiO_2_ film, and the generation of internal stress in the film is an important reason for its delamination. After adjusting the argon gas filling amount, the defects in the SEM image of the film were significantly reduced, and the film no longer had a peeling phenomenon.

The control of film stress is the primary method to reduce the phenomenon of film peeling. Especially for a multilayer film on an ultra-thin substrate, the substrate itself is prone to deformation, so the stress generated by the film layer has a more obvious impact on it. The negative effects of film stress can be reduced by adjusting the coating pressure, the temperature of the evaporation substrate, and the oxygen pressure content of some oxide materials. At the same time, it is also possible to choose the ratio of high and low refractive index materials when designing the film system, except that the spectral conditions are satisfied. At the same time, the problem of stress generation can also be optimized. Avoid film stress due to grain boundary void shrinkage, grain boundary relaxation, vacancy annihilation, impurity merger, etc.

## 5. Conclusions

In this paper, the film peeling of Nb_2_O_5_ and SiO_2_ multilayer dielectric films is solved by analyzing the test results of the spectrum, SEM, and stress analyzer for many experiments. The stress of SiO2 film decreases first and then increases with the increase of pressure, first increases and then decreases with the increase of oxygen partial pressure within a certain range, and increases with the increase of substrate temperature. The thermal stress, internal stress, and external stress of the film stress are analyzed and improved in detail. Through the stress analysis and spectral analysis of large-angle ultra-thin short-wave pass filter, a short-wave pass filter with high transmittance, low stress, and not easy to peel off is prepared. A wide-angle short-wave filter based on ultra-thin silicate glass is designed and fabricated according to the equivalent layer matching theory and boundary conditions. The sample meets various test requirements.

## Figures and Tables

**Table 1:** Filter film parameters requirements.

Incident Angle/°	Wavelength/nm	Transmittance/%
0	410~810	>96
	850~1100	<1
35	410~775	>96
	830~1100	<1

**Table 2:** Thin-film structure and film thickness.

Num	Thickness	Num	Thickness	Num	Thickness	Num	Thickness	Num	Thickness
	Sub	15	0.58L	30	0.37H	45	0.38L	60	2.10H
1	0.48L	16	2.11H	31	0.48L	46	0.44H	61	0.36L
2	0.51H	17	2.09L	32	2.03H	47	0.39L	62	0.44H
3	0.26L	18	1.94H	33	0.46L	48	2.14H	63	0.40L
4	1.11H	19	2.03L	34	0.39H	49	0.36L	64	2.16H
5	0.21L	20	1.87H	35	0.44L	50	0.46H	65	0.51L
6	0.55H	21	0.59L	36	2.03H	51	0.34L	66	0.33H
7	2.16L	22	0.26H	37	0.40L	52	2.10H	67	0.53L
8	1.96H	23	0.60L	38	0.39H	53	0.31L	68	2.08H
9	2.05L	24	1.98H	39	0.42L	54	0.50H	69	0.46L
10	1.92H	25	0.57L	40	2.11H	55	0.34L	70	0.37H
11	2.04L	26	0.29H	41	0.43L	56	2.12H	71	0.45L
12	2.05H	27	0.59L	42	0.41H	57	0.39L	72	1.89H
13	0.62L	28	2.01H	43	0.43L	58	0.43H	73	0.87L
14	0.34H	29	0.47L	44	2.10H	59	0.35L		Air

## Data Availability

The data in this research is available from the corresponding author upon reasonable request.

## References

[1] Li W.Q., Mu C.Y. (2021). Charging effects of SiO_2_ thin film on Si substrate irradiated by penetrating electron beam. Micron.

[2] Yu L.F., Ye Y.T., Wu J.P. (2012). Defect detection and control of a laser conditioning system for large diameter optical film. Laser Technol..

[3] Amato A., Cagnoli G., Granata M., Sassolas B., Degallaix J., Forest D., Michel C., Pinard L., Demos N., Gras S. (2021). Optical and mechanical properties of ion-beam-sputtered Nb_2_O_5_ and TiO_2_-Nb_2_O_5_ thin films for gravitational-wave interferometers and an improved measurement of coating thermal noise in Advanced LIGO. Phys. Rev. D.

[4] Zhang Y., Zhang Y., Ling N., Xu H. (2005). Research on Finite Element Analysis of Thin Film Residual Stress. Prog. Laser Optoelectron..

[5] Zhang B., Chen T., Xiong Y., Wang D., Wang C. (2010). Research on the design and plating technology of mid-wave infrared short-wave pass filter. Vac. Cryog..

[6] Zhao X., Shi J., Guo H. (2006). Short-wave pass filter film system design. Appl. Opt..

[7] Leng J., Xue W., Yu Z., Lu W., Wang H., Zhang D. (2011). On-line measurement and research of TiO_2_ and SiO_2_ film stress. J. Vac. Sci. Technol..

[8] Rani R.A., Zoolfakar A.S., O’Mullane A.P., Austin M.W., Kalantar-zadeh K. (2014). Thin films and nanostructures of niobium pentoxide: Fundamental properties, synthesis methods and applications. J. Mater. Chem. A.

[9] Hoffmann M.R., Martin S.T., Choi W., Bahnemann D.W. (1995). Environmental application of semiconductor photocatalysis. Chem. Rev..

[10] Wang X., Fang W., Liu F. (2020). Material properties of MoOx thin film prepared by magnetron sputtering. Acta Sol. Energy.

[11] Stoney G.G. (1909). The tension of metallic films deposited by electrol-ysis. Soc. Lond. Ser. A.

[12] Maschietto M., Dal Maschio M., Girardi S., Vassanelli S. (2021). In situ electroporation of mammalian cells through SiO_2_ thin film capacitive microelectrodes. Sci. Rep..

[13] Park C., Lee J., Kwon K.S., Cho K.Y., Kim J. (2021). Fabrication of electrochromic devices by laser patterning of spin-sprayed transparent conductive Ga: ZnO films. Ceram. Int..

[14] Zhang L., Xiao X., Qi H., Huang Y., Qin H. (2022). Quantitative and nondestructive determination of residual stress for SiO_2_ thin film by laser-generated surface acoustic wave technique. Meas. Sci. Technol..

[15] Harashima T., Kinoshita J.I., Kimura Y., Brugnera A., Zanin F., Pecora J.D., Matsumoto K. (2005). Morphological comparative study on ablation of dental hard tissue at cavity preparation by Er: YAG and Er, CR: YSGG lasers. Photomed. Laser Surg..

[16] Ishikawa I., Aoki A., Takasaki A.A. (2008). Clinical application of erbium: YAG Laser in periodontology. J. Int. Acad. Periodontol..

[17] Teer G. (1989). Technical note: A magnetron sputter ion-plating system. Surf. Coat. Technol..

[18] Sproul W.D., Rudnik P.J., Graham M.E., Rohde S.L. (1990). High rate reactive sputtering in an opposed cathode closed-field unbalanced magnetron sputtering system. Surf. Coat. Technol..

[19] Sulieman M. (2005). An overview of the use of lasers in general dentist practice: Laser physics and tissue interactions. Dent. Update.

[20] Bhattarai M.K., Mishra K.K., Instan A.A., Bastakoti B.P., Katiyar R.S. (2019). Enhanced energy storage density in Sc^3+^ substituted Pb(Zr_0.53_Ti_0.47_)O_3_ nanoscale films by pulse laser deposition technique. Appl. Surf. Sci..

[21] McNerny D.Q., Viswanath B., Copic D., Laye F.R., Prohoda C., Brieland-Shoultz A.C., Polsen E.S., Dee N.T., Veerasamy V.S., Hart A.J. (2014). Direct fabrication of graphene on SiO_2_ enabled by thin film stress engineering. Sci. Rep..

[22] Weiner G.P. (2004). Laser dentistry practice management. Dent. Clin. N. Am..

[23] Lee B.J., Seo D.W., Choi J.W. (2021). Analysis of the influence of disk and wafer rotation speed on the SiO_2_ thin-film characteristics in a space-divided PE-ALD system. J. Korean Phys. Soc..

[24] Loevschall H., Arenholtd-Bindslev D. (1994). Effect of low level diode laser irradiation of human oral mucosa fibroblasts in vitro. Lasers Surg. Med..

[25] Noble P.B., Shields E.D., Blecher P.D., Bentley K.C. (1992). Locomotory characteristics of fibroblasts within a three-dimensional collagenlattice: Modulation by a helium/neon soft laser. Lasers Surg. Med..

[26] Asencio Arana F., Garcia F.V., Molina Andreu E., Vidal M.J., Martinez S.F. (1992). Endoscopic enhancement of the healing of high risk colon anastomoses by low-power helium-neon laser. An experimental study. Dis. Colon Rectum..

[27] Pourreau-Schneider N., Ahmed A., Soudry M., Jacquemier J., Kopp F., Franquin J.C., Martin P. (1990). Helium-neon laser treatment transforms fibroblasts into myofibroblasts. Am. J. Pathol..

[28] Neiburger E.J. (1995). The effect of low-power lasers on intraoral wound healing. N. Y. State Dent. J..

[29] Kurumada F. (1990). A study on the application of Ga-As semiconductor laser to endodontics. The effects of laser irradiation on the activation of inflammatory cells and the vital pulpotomy. Ohu Daigaku Shigakushi.

[30] Kitsmaniuk Z.D., Demochko V.B., Popovich V.I. (1992). The use of low energy lasers for preventing and treating postoperative and radiation-induced complications in patients with head and neck tumors. Vopr. Onkol..

[31] Lira-Cantu M., Krebs F.C. (2006). Hybrid solar cells based on MEH-PPV and thin film semiconductor oxides (TiO_2_, Nb_2_O_5_, ZnO, CeO_2_ and CeO_2_–TiO_2_): Performance improvement during long-time irradiation. Sol. Energy Mater. Sol. Cells.

[32] Pond B.J., DeBar J.I., Carniglia C.K., Raj T. (1989). Stress reduction in ion beam sputtered mixed oxide films. Appl. Opt..

[33] Windischmann H. (1991). Intrinsic stress in sputtered thin films. J. Vac. Sci..

[34] Shao S. (2002). Study of the Origin Mechanism and Controlling Method of Stress in Thin Films. Ph.D. Thesis.

[35] Martin P.J., Netterfield R.P., Sainty W.G. (1984). Modification of the optical and structural properties of dielectric ZrO_2_ films by ion-assisted deposition. J. Appl. Phys..

[36] Qin Y.W. (2012). Film thickness measurement based on optical coherence tomography. Laser Technol..

[37] Tokas R.B., Sahoo N.K., Thakur S., Kamble N.M. (2007). A comparative morphological study of electron beam co-deposited binary optical thin films of HfO_2_: SiO_2_ and ZrO_2_: SiO_2_. Curr. Appl. Phys..

[38] Ren H., Zeng Q., Liang X.H. (2012). Characteristics of Nd: Y3Al5O12 thin film prepared by electron beam evaporation deposition. Laser Technol..

[39] Schell-Sorokin A.J., Tromp R.M. (1990). Mechanical stresses in (sub) monolayer epitaxial films. Phys. Rev. Lett..

[40] Thornton J.A., Hoffman D.W. (1989). Stress-related effects in thin films. Thin Solid Film..

[41] Reicherd W., Mcco R., Mack S.A. (2000). Production of thin film optical coatings with predetermined stress levels. Imaging Technology and Telescopes.

[42] Ghang Y., Jin C., Li C., Deng W., Jin J. (2014). ArF excimer laser induced damage on high reflective fluoride films. Laser Technol..

